# Interventionist videothermometry: a new model of cardiac ischemia evaluation

**DOI:** 10.1186/s12917-020-02358-8

**Published:** 2020-05-19

**Authors:** Tomas Ottoni Barroso da Silva, Leonardo Waldstein de Moura Vidal, Paula Gebe Abreu Cabral, Matheus Roberto da Mota Costa, Silvia Marcela Ruiz Cadena, Marcelo Borges dos Santos Junior, Fernanda Antunes, André Lacerda de Abreu Oliveira

**Affiliations:** Darcy Ribeiro North Fluminense State University, Alberto Lamego Avenue, Campos dos Goytacazes, 2000 Brazil

**Keywords:** Temperature, Thoracic surgery, Thermometry, Heart

## Abstract

**Background:**

The purpose of the present study was to evaluate, through videothermometry, the temperature variation in the hearts of rabbits, that underwent induced myocardial ischemia and reperfusion.

**Results:**

A total of 20 female rabbits were divided into two groups: a treated group and a sham group, the treatment group underwent 5 min of cardiac arrest and reperfusion, using the inflow occlusion technique. Throughout the experiment, the animals were monitored by videothermometry, observing the thermal variations of the myocardial tissue. During the experiment, at different times, blood gas tests and tests to evaluate the lactate concentrations were performed. At the end of the experiment, each heart was submitted to histopathological evaluation. In the treated group, there was a reduction in temperature of the myocardial tissue during the circulatory arrest compared to the sham group. Additionally, a colder area next to the caudal vena cava ostium and the right atrium was observed. Notably, despite the 5 min of cardiac arrest in the treated group, both the lactate and bicarbonate levels were maintained without significant variation. However, there was an increase in PaCO2 and pH reduction, featuring respiratory acidosis. In relation to the histopathological study, the presence of hydropic degeneration in the myocardium of animals in the treated group was observed.

**Conclusions:**

Based on these results, the videothermometry was efficient in identifying the range of myocardial tissue temperature, suggesting that the first areas to suffer due to cardiac arrest were the caudal vena cava ostium and the right atrium. However, in regard to the angiographic coronary thermography, the study was not feasible due to the small size of the coronary. There was no variation between the groups regarding the presence of myocardial infarction, myocardial congestion, myocardial edema and myocardial hemorrhage.

## Background

Interest in a new method for the early diagnosis of the presence of myocardial ischemia, especially during the surgical treatment of acquired and congenital heart disease, has been increasing in veterinary medicine.

Currently, the main methods available to assess the presence of myocardial ischemia in pets during the perioperative period of cardiac surgery are continuous electrocardiography [1] and transesophageal echocardiography [2]. Electrocardiographic changes and a reduction of systolic indices in response to an established ischemic process are expected [2, 3].

The possibility of the early detection of cardiac ischemia areas makes videothermometry a promising tool, that may facilitate the prognosis of animals undergoing cardiac surgery, once the identification of these injuries by the method occurs prior to electrocardiographic abnormalities or the reduction of systolic indices. In medicine, thermometry is used with the same purpose (e.g., for detection of intraoperative coronary obstruction) [4] and for atherosclerotic cardiovascular disease prior to the development of clinical signs of inflammation and acute myocardial infarction [5]. In veterinary medicine, regarding livestock, thermometry is widely used to detect temperature variations, indicating the presence of local inflammatory process. However, there are no data regarding the use of thermometry during the cardiac surgery perioperative period.

Thus, the present study contributes, in an unprecedented manner, an evaluation of ischemic heart disease in veterinary medicine, offering the potential for increasing subsidies to better understand the evolution of the cardiac ischemic process during cardiac circulatory arrest. To verify the applicability of videothermometry in the detection of cardiac ischemia, a model was used to study the inflow occlusion technique, which leads to temporary cardiac ischemia.

The objectives of the present study are to evaluate, using videothermometry, the temperature variations in the hearts of rabbits, that underwent induced myocardial ischemia and reperfusion, as well as evaluate the blood gas parameters (pH, PaCO2, HCO3) and lactate concentrations at different times at 0, 5 and 10 moment. The term “Thermometry”, indicates a diagnostic method that examines specific fields, observing the skin microcirculatory activity and its distribution on the skin surface, to investigate the thermal nature produced by the disease. In the present study, the nomenclature “videothermometry” refers to surgical scanning in real time, analyzing thermal changes of vessels, tissues and organs in search of metabolic disorders. The hypothesis is that videothermometry is effective in detecting areas of cardiac ischemia in the hearts of rabbits, that underwent cardiac ischemia and reperfusion.

## Results

**Fig. 1:**
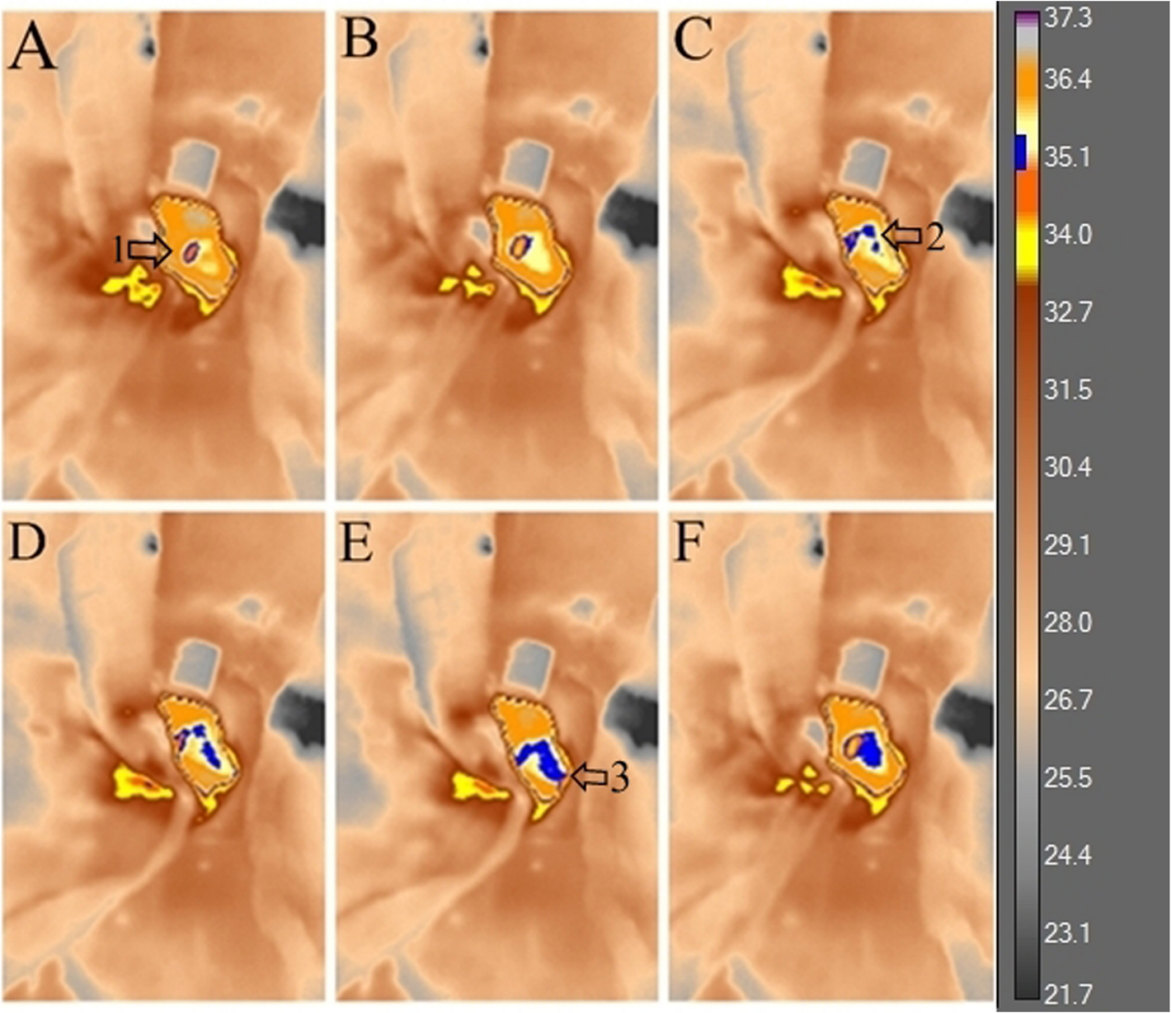
Videothermometry infrared in normal heart. The figure indicates the variation of myocardial tissue temperature, which is represented by the blue isotherm color, indicating the heat variation inside the heart (**a**), (**b**), (**c**), (**d**), (**e**), (**f**). The numbers indicated by the arrows, 1, 2, 3; represent the caudal vena cava, the right atrium and the right ventricle, respectively. The blue isotherm increases indicating a gradual decrease in temperature (from 36.4 °C to 35.1 °C) through the temperature indicator bar on the right side. This decrease in temperature indicates a decrease in blood flow. UEA / UENF, 2015

During the videothermometry, we observed a change in myocardial tissue temperature, visualized through the blue isotherm, indicating the temperature variations inside the heart of the treated group animals during the M0 moment. The isotherm is a color applied to the image, within a specific temperature range to demonstrate a particular appearance. In this case, this aspect is the heat variation inside the heart, indicating the movement of the blood flow from the caudal vena cava to the right atrium and right ventricle (Fig. 1).

**Fig. 2:**
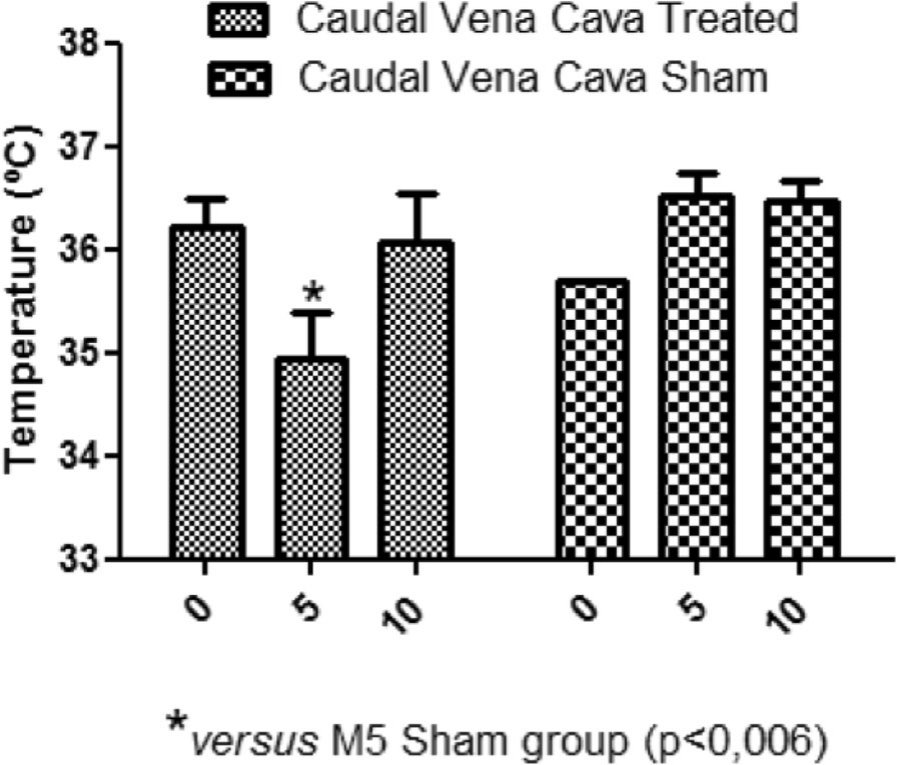
Caudal vena cava temperature. Average of the caudal vena cava temperatures at different moments. M5 treated group temperature reduction compared to the sham group

**Fig. 3:**
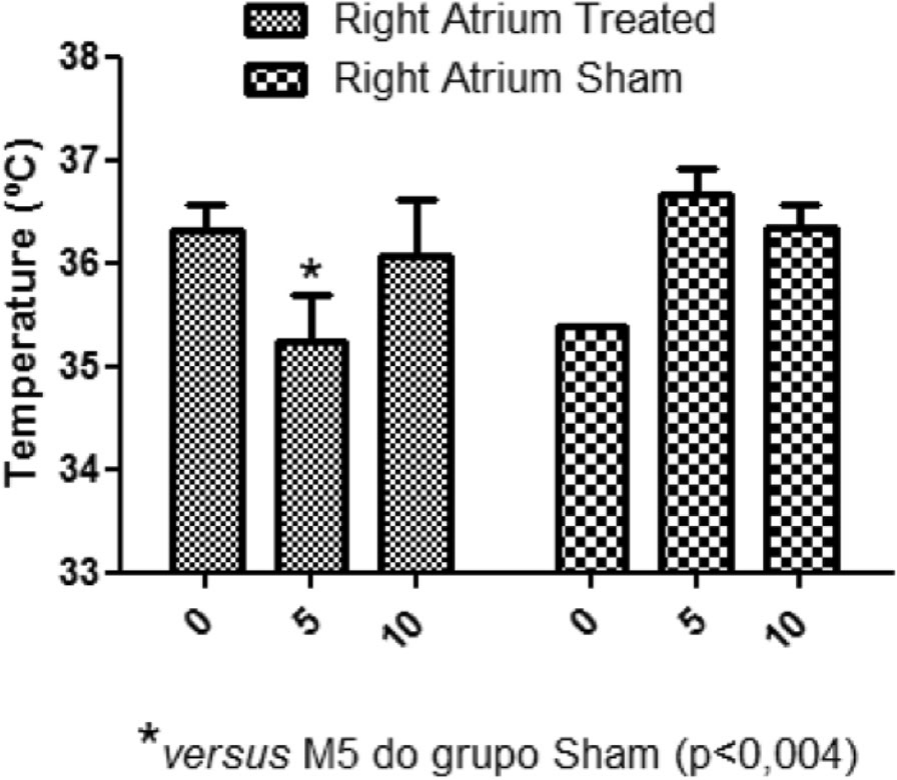
Right atrium temperatures. Average of the right atrium temperatures at different moments. M5 treated group temperature reduction compared to the sham group

**Fig. 4:**
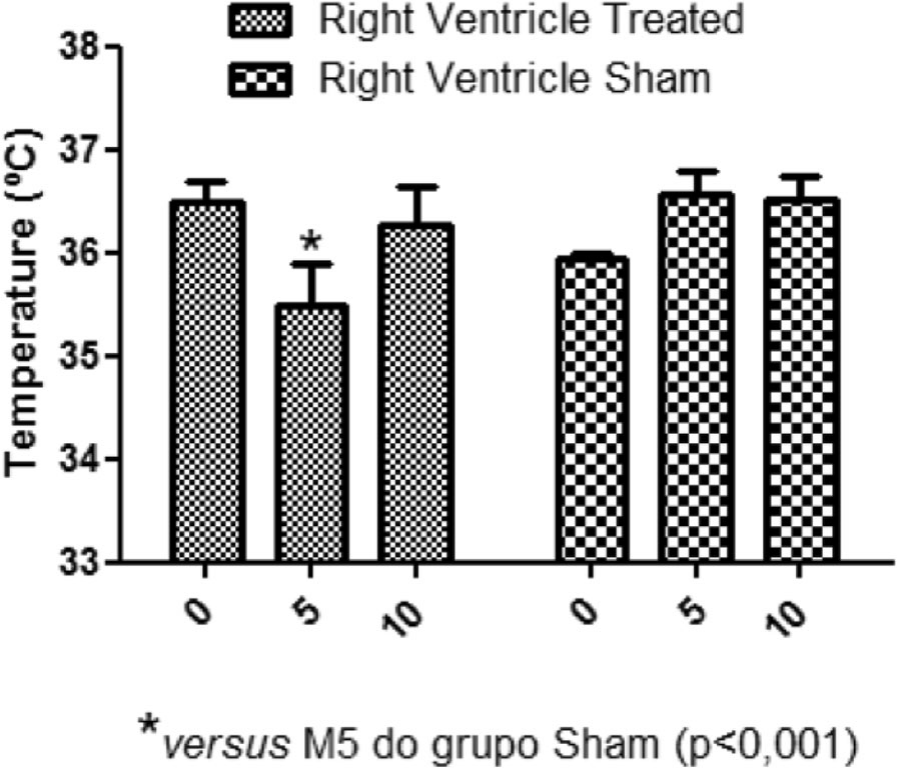
Right ventricle temperature. Average of the right ventricle temperatures at different moments. M5 treated group temperature reduction compared to the sham group

In the present study, during moment 5 (M5), there was a reduction of the caudal vena cava, the right atrium and right ventricle temperature of the treated group animals, compared to the same moment of the sham group (Figs. 2, 3 and 4).

**Fig. 5:**
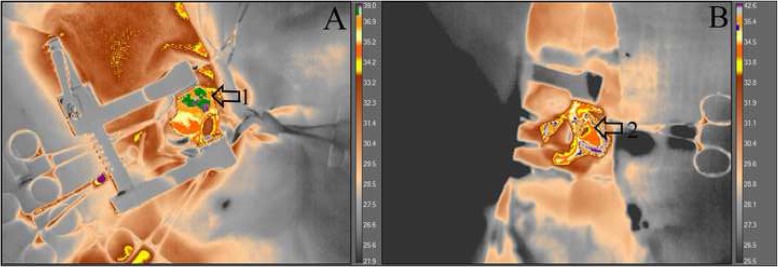
Videothermometry infrared during respiratory arrest and cardiac ischemia periods. The figure represents two groups; (**a**) sham group during ventilatory arrest period M5 and (**b**) treated group during cardiac ischemia period M5. Observe the respective temperature bars and the heat variations in the myocardial tissue according to the colors. In (**a**) a green isotherm was used and the temperature between 36.9 °C and 39 °C is noted. In (**b**) a blue isotherm was used and the temperature ranges between 32 °C and 34.5 °C. Arrows 1 and 2, indicate the location of the heart, in the caudal vena cava and right atrium areas, a brown spot is evident, meaning a cooler region with the temperature approximately 32 °C. The treated group, demonstrated a considerable reduction in temperature, indicating a significant decrease when myocardial ischemia occurs. UEA / UENF, 2015

**Fig. 6:**
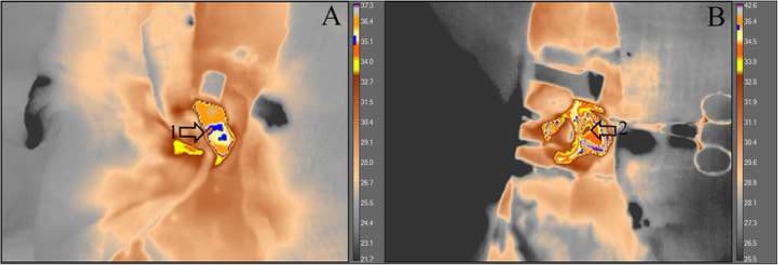
Videothermometry infrared in a normal heart and in a ischemic heart. The images represent the treated group, at moments M0 (A) and M5 (B). In the moment 0 (**a**) note the blue isotherm, indicating a temperature approximately 35.1 °C and 36.4 °C (1). In the moment 5 (**b**) note the absence of the blue isotherm and the presence of colors which indicates a temperature between 31.1 °C and 32.8 °C (2). Through this sequence of images, it is possible to evaluate the temperature reduction that occurred between the beginning and the end of the procedure ORCID is present in the manuscript and was captured as provided in the manuscript. Please confirm if action taken is appropriate.

**Fig. 7:**
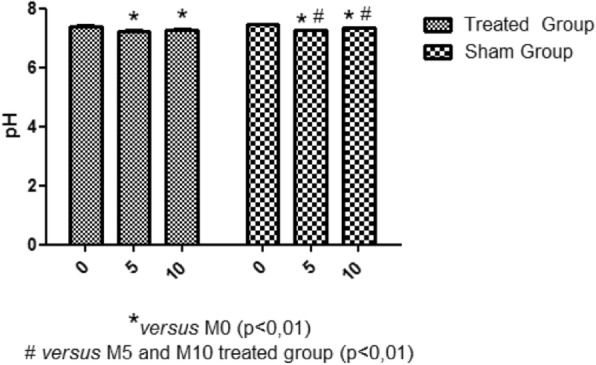
pH variations. Acidemia presented by rabbits in treated group. Reduction in pH during de M5 and M10 moments, when compared with M0

**Fig. 8:**
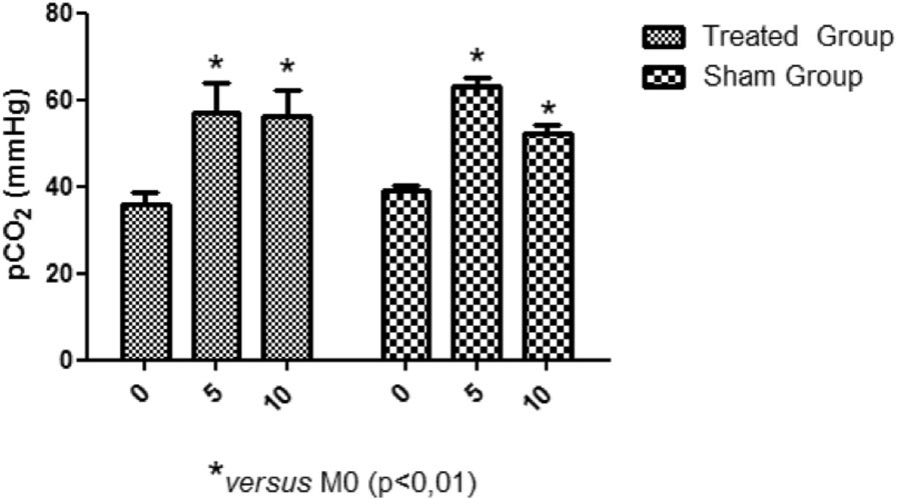
pCO2 variations. Increased PCO2 presented by rabbits in the treated group during M5 and M10 when compared with M0 moment

There was a reduction of heart temperature between the treated group and the sham group during M5 moment. This change can be visualized with green isotherm, indicating the myocardium temperature variation (sham group) (Fig. 5). Additionally, a temperature reduction of the myocardial tissue, was observed during the circulatory arrest period in the treated group (Fig. 6). The average temperature in the animals from the treated group during the experiment at moment 0 was approximately 35.1 °C - 36.4 °C and at moment 5 approximately 31.1 °C - 32.8 °C, representing temperature reduction of approximately 2.3 °C to 5.3 °C. While the untreated group had temperatures between 36.4 °C - 39 °C. Regarding hemogasometric evaluation, the following parameters were analyzed: pH, bicarbonate and PaCO2. The values obtained at moment M0 were considered as the control values in the treated group. The pH values significantly decreased during the ischemia and reperfusion periods, indicating the presence of acidosis (Fig. 7). The PaCO2 values increased during ischemia/reperfusion (Fig. 8). This result indicates that acidosis has a respiratory origin.

**Fig. 9:**
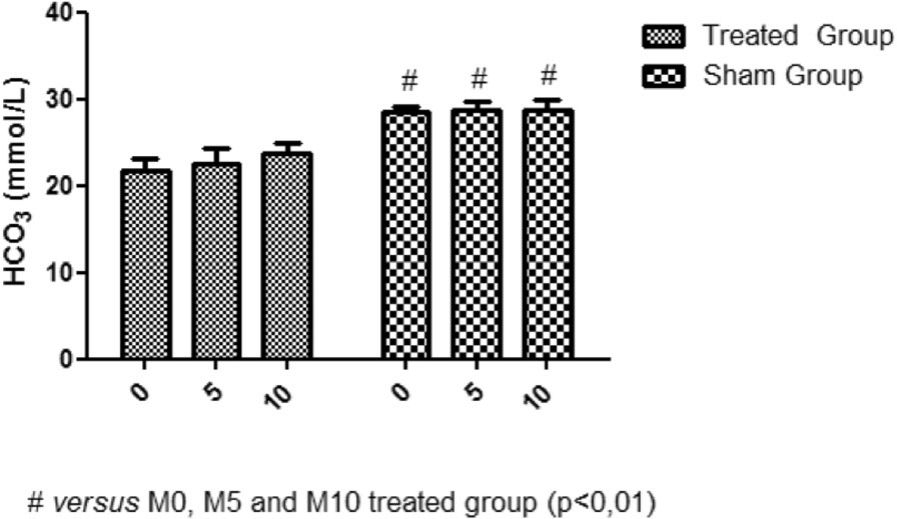
Bicarbonate variation. No variation of the bicarbonate concentration between moments M0, M5 and M10

**Fig. 10:**
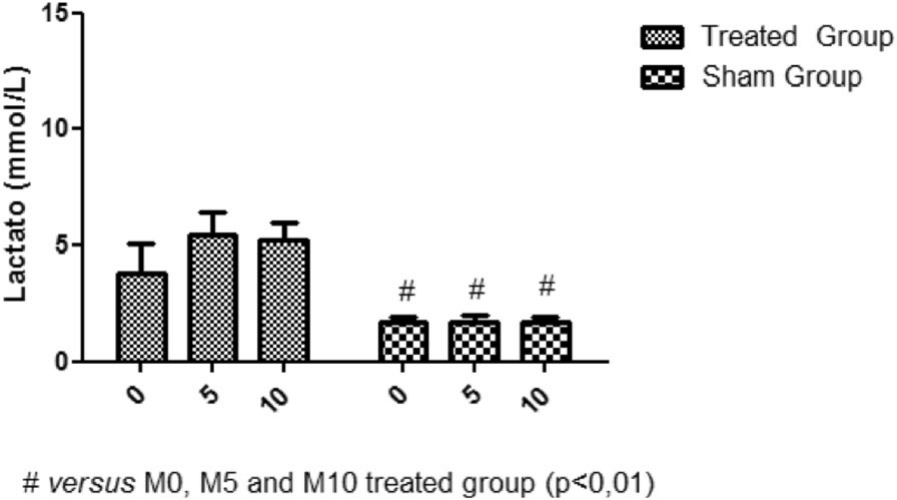
Lactate variation. No change in lactate levels between M0, M5 and M10 moments

There was no statistically significant variation in both bicarbonate and lactate values, when comparing the M5 and M10 moment to the M0 moment (Fig. 9; Fig. 10).

**Fig. 11:**
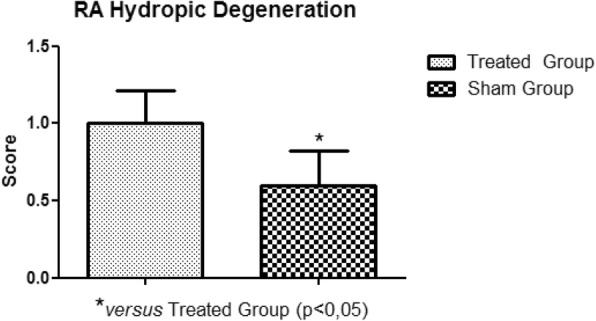
Right Atrium Hydropic Degeneration. Presence of hydropic degeneration in the right atrium of the treated group rabbits

**Fig. 12:**
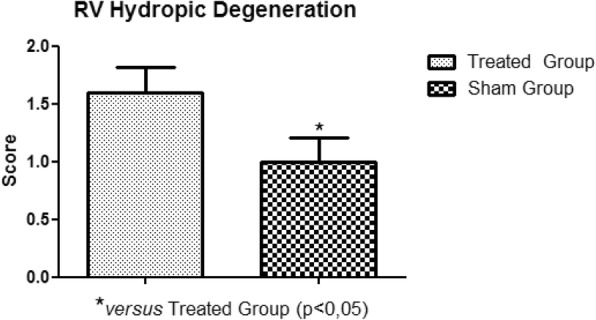
Right Ventricle Hydropic Degeneration. Presence of hydropic degeneration in the right ventricle of the treated group rabbits

**Fig. 13:**
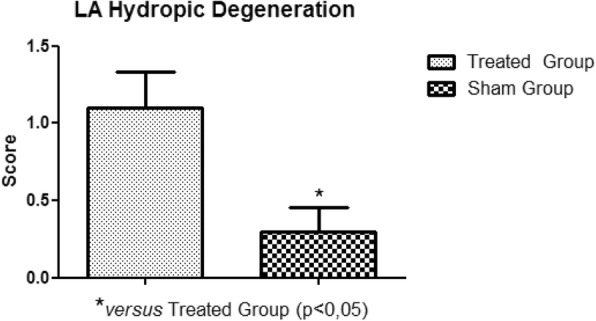
Left Atrium Hydropic Degeneration. Presence of hydropic degeneration in the left atrium of the treated group rabbits

**Fig. 14:**
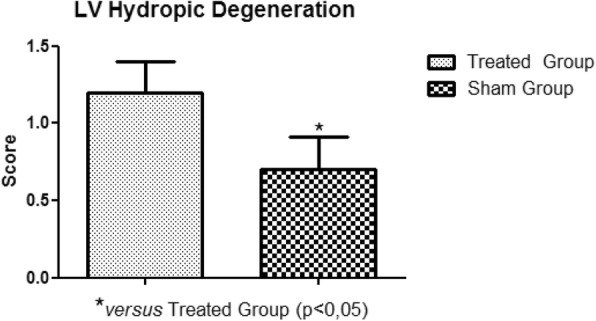
Left Ventricle Hydropic Degeneration. Presence of hydropic degeneration in the left ventricle of the treated group rabbits

In the histopathological study of the samples collected, no significant variations were observed between the treated and sham groups, in relation to areas of edema, myocardial infarction, congestion and hemorrhage. The significant presence of hydropic degeneration observed in the samples was characteristic of the animals of the treated group. The degeneration was established in the right atrium, right ventricle, left atrium and left ventricle (Fig. 11, Fig. 12, Fig. 13, Fig. 14).

## Discussion

During heart surgery, the thermal gradient generated by the coronary filling stream, noninvasively enables the design of the arteries and formation of an angiographic thermal image. Thus, in the perioperative period, this method is a great aid in procedures involving the coronary arteries, including testing the viability of grafts and anastomoses, and it provides the speed of coronary blood flow, identifying potential areas of obstruction [4, 6, 7]. Although videothermometry was used in an unprecedented manner during cardiac ischemia study, we could not observe or define the coronary arteries of the rabbit heart because of the rabbit heart size. However, it was possible to follow the variation of heat throughout the myocardial tissue, demonstrating a significant reduction in temperature during circulatory arrest when compared to the sham group during the same time. This heat reduction occurred because of the obstruction of the blood flow to the heart, leading to hypoperfusion and thus a reduced heat emission by cardiomyocytes. The heat emission by the myocardium is determined by both cardiac blood flow and the metabolism [8].

We propose that the induction of circulatory arrest was responsible for hypothermia at the M5 moment in the treated group. The relationship between the temperature of the patient in the perioperative period and the time of circulatory arrest are closely related to the application of inflow occlusion technique, being observed hypothermia in patients who undergoing this technique [9]. However, in some cases a lower patient temperature was observed during the moment before the circulatory arrest. This lower temperature at the time prior to cardiac arrest was justified by anesthesia [10].

The videothermometry can be affected by external factors, such as the ambient temperature where the exam is performed. Therefore, a controlled environment is essential to ensure the reliability of videothermographic image [11]. In the present study, the operating room environment was maintained at a conditioning of 18 °C and was not observed any interference in ambient temperature to obtain the thermal images.

Episodes of acid-base imbalance are commonly observed during heart surgery, especially when there is occlusion of the venous return, causing tissue hypoxia. This situation occurs in procedures involving the inflow occlusion technique [12]. The diagnosis of acid-base disorders is based on pH, PCO2 and the bicarbonate concentrations in the blood, where an increase of H+ ions is indicative of acidosis and a reduction of the same ions, as well as alkalosis [13]. The analysis of PCO2 and bicarbonate concentrations helps to determine the precise cause of the pH variation [14]. With each Celsius degree variation, the pH undergoes a change of 0.15 units, indicating that temperature variations significantly affect the pH in the blood [15, 16].

In the present study, during the M5 and M10 moments of the treated group, there was a decrease in blood pH, but we cannot attribute this reduction to changes in body temperature alone. This reduction occurred during conditions of anaerobic metabolism [17, 18]. In the treated group, this condition was obtained by cardiac arrest.

Kwasnicka et al. (2000) and Vale et al. (2016) observed a reduction in arterial pH of rats undergone 5 min of venous return occlusion, using the inflow occlusion technique and subsequent recirculation, consistent with the results obtained in the present study. The blood recirculation in the treated group was not sufficient to reverse the acidosis, which was established after occlusion of the caudal vena cava, cranial vena cava and azygos vein.

The PaCO2 significantly increased in response to the period of circulatory arrest due to reduced supply of oxygen, while remaining high even after blood recirculation, consistent with the results of Vale et al. (2016).

There was no significant change in bicarbonate concentrations with 5 min of cardiac arrest, confirming the findings of Kwasnicka et al. (2000), who found no variation in bicarbonate concentrations in a study of dogs that underwent 5 min of cardiac and reperfusion arrest.

There was no statistically significant change in lactate levels. However, the establishment of the inflow occlusion technique leads to a significant increase in lactate at the time of circulatory arrest because of the transition to anaerobic metabolism and not even the recirculation period is sufficient to normalize lactate values [18]. The ischemia period was not sufficient to cause a significant variation in the lactate concentration between the treated group and the sham group. The tissue becomes acidic because of the accumulation of lactate. The pH falls to approximately 6.6 during the first 10 min and equilibrates at 5.8 after 50 min of ischemia [19].

Hydropic degeneration is the first change observed in cells presenting degenerative process, leading to accumulation of intracellular fluid. Perhaps due to the short period of ischemia and reperfusion, the other lesions, such as myocardial edema, myocardial infarction and myocardial congestion, although present, did not have a significant variation between the treated and sham groups. The total tissue water increases 21% after 2 min and 43% after 20 min of reperfusion [20]. Such increases may indicate the need for a longer reperfusion period to obtain, for example, myocardial edema variation.

## Conclusions

In conclusion, videothermometry was efficient in identifying the variation of the myocardial tissue temperature. Although the absence of significant myocardial infarction in both structures, it was possible to observe a lower temperature area next to the caudal vena cava ostium and the right atrium, during the cardiac ischemia period, in the treated group. This observation may indicate that the previously mentioned structures are the first areas to suffer with the processes related to cardiac ischemia.

With the establishment of the circulatory arrest and reperfusion period, the presence of hydropic degeneration in the right atrium, right ventricle, left atrium and left ventricle was observed. Other lesions, such as myocardial edema, myocardial congestion and myocardial infarction, although present, did not present a statistically relevant variation when compared to the sham group. We recommend increasing the ischemia and reperfusion period for the significant identification of lesions in the histopathological study.

We also conclude that in the rabbit heart, angiographic thermography studies of the coronary arteries are not feasible due to the small size of the structures, requiring the use of a larger experimental animal species for this purpose.

## Methods

The present study was conducted in full compliance with all applicable research ethics and animal welfare regulations, under a general stranding response authorization by the Animal Use Ethics Committee (CEUA) of the Northern Fluminense State University Darcy Ribeiro, under the protocol number: 171/2012, according to brazilian federal law n ° 11794/08.

The *Oryctolagus cuniculus* rabbits were obtained from Federal University of Viçosa, vivarium sector. The animals were kept at the experimental unit in group cages with no more than 2 rabbits in each space with feeding system ad libitum. The animals were contained manually and received a pre-anesthetic protocol based on acepromazine, at a dose of 0.5 mg/kg/ IM, associated with morphine sulfate at a dose of 4 mg/kg/IM. After obtaining the desired anesthetic effect, trichotomy was performed on the left and right pinna, followed by catheterization of the marginal vein of the left external ear. This access enabled saline 0.9% infusion through syringe pump with a controlled dose of 15 ml/hr. Catheterization of the central artery of the external right ear was performed, enabling the collection of blood samples for hemogasometric and lactate analysis. Syringes for blood collection, were flushed with heparin sodium, and the material was evaluated immediately after collection. Subsequently, the trichotomy of the ventral area of the neck and right thoracic region was performed, enabling, respectively, tracheostomy and surgical access. Finally, a propofol bolus was intravenously administered at a dose of 10 mg/kg, and then tracheostomy and intubation were performed, enabling the realization of manual ventilation. Tracheal tube number 3.0 was connected to an inhalation anesthesia machine through the Baraka system. The animals were maintained under anesthesia with isoflurane inhalation, in a universal vaporizer.

The temperature for conditioning the operating room was 18 °C. All rabbits were subjected to control blood collection (M0) and hemogasometric evaluation, by a portable blood analyzer, to obtain the pH, PaCO2 and HCO3. The same analyzer provided lactate values. A thermal mattress was not used during the procedure. The temperature of the animals was monitored through videothermometry.

The animals were divided into two groups: the treated group and the sham group. The treated group consisted of 10 animals that underwent right lateral thoracotomy in the fourth intercostal space, followed by dissection of anatomical planes to access the chest cavity, and held the dorsal retraction of the lung lobes to expose the caudal mediastinum. After the incision in the caudal mediastinum and careful dissection of the structures, the caudal vena cava, cranial vena cava and azygous vein were identified and individualized, followed by dorsal retraction of the phrenic nerve. The pericardium was incised along the cardiac axis. In sequence, the Satinsky clamps were positioned to respectively occlude the caudal vena cava, the azygous vein and the cranial vena cava. A 30 s period of hyperventilation was performed immediately prior to occlusion. The clamping of vessels by using calipers was maintained for a period of 5 min, and subsequently a blood sample was collected for hemogasometric and lactate evaluation (M5). After 5 min of inflow occlusion, the Satinsky clamps were released, enabling cardiac reperfusion and establishing 5 min of recirculation. After this period, a new blood sample was collected for hemogasometric and lactate concentration evaluation (M10). Then, the rabbits underwent a painless death.

The sham group consisted of 10 animals who underwent the same methodology regarding the surgical access to the animals of the treated group. The pericardial incision along the cardiac axis and careful dissection and identification of the caudal vena cava, cranial vena cava and azygous vein was established in 5 min without any ventilation and circulatory arrest. Subsequently, new blood gas analysis and lactate concentration was performed (M5). Then, the animals were manually ventilated for 5 min, followed by blood sample collection to evaluate the blood gas and lactate concentration (M10). In sequence, the painless death of the rabbits was performed.

For the generation of videothermometric images, we used the MART station, which consists of a computerized work island, linked to an infrared image generator through a lance, which can be adjustable by distance. The distance is important because it enables the patient, lying on a surgical stretcher at a 1 m away to frame the evaluation field. The MART station is placed far from the surgical field, leaving free space for the normal flow of the surgical room, and providing a thermographer (the person performing and editing images through dedicated MART 1.0 software). These professional processes and returns real-time images during surgery. The MART station captures multi and hyperspectral images, performs mapping of multimodal thermal texture, sensor fusion and three-dimensional image. This system is radiation free for reading and medical diagnosis.

The following periods were set for videothermometric analysis regarding the treated group: M0 (time just prior to cardiac arrest); M5 (period of time which the animal was subjected to circulatory arrest); and M10 (period of time which the animal was subjected to reperfusion). The heart temperature was constantly evaluated in each moment. At each period, three temperature values were obtained, and an average of the values were calculated for each moment.

The periods set for videothermometric analysis regarding the sham group were as follows: M0 (period of time immediately before the respiratory arrest); M5 (period of time which the animal was underwent to respiratory arrest); and M10 (period of time which the animal was subjected to the return of ventilation). The heart temperature was constantly evaluated in each moment. Three temperature values, and average values were calculated for each moment.

At the end of the trial, the hearts were collected from animals in both groups, treated and sham, for histopathological analyses to evaluate cell lesions, such as edema, hydropic degeneration, congestion, myocardial infarction and hemorrhage. The histopathological analyses were realized at the Veterinary Anatomopathological Diagnostic Laboratory in the Animal Pathology Sector, which was focused on histological variations among the right atrium, the right ventricle, the left atrium, the left ventricle and the interventricular septum.

Respecting the ethical principles of animal experimentation, immediately after the experiment, the animals were euthanized. The animals were subjected to deep general anesthesia with propofol administration, and subsequently administered 10 ml of 19.1% potassium chloride via intracardiac injection.

In the statistical study of hemogasometry and lactate concentration, statistical analysis within each group was performed by ANOVA, followed by Tukey’s post-test to compare the means between the moments (M0, M5 and M10). The comparisons between the groups were performed by Student’s t-test. The significance level established for the statistical test was (*p* < 0.05).

In relation to videothermometry, the statistical analysis within each group was performed using Two Way Variance Analysis (ANOVA), followed by Mann-Whitney’s post-test test to compare the means between the moments (M0, M5, and M10). The significance level established for the statistical test was (*p* < 0.05).

## Data Availability

The datasets supporting the conclusions of this article are included within the article. The raw data are available from the corresponding author on reasonable request.

## Abbreviations

IM: Intramuscular
MART: Metabolic Activity in Real Time
pH: Arterial blood acidity or alkalinity
PaCO2: Partial pressure of carbon dioxide in arterial blood
HCO3: Bicarbonate ions in arterial blood
